# Supplementary material for: Current Utilization and Research Status of the Herbal Medicine Guibi-Tang and Its Variants for Cognitive Impairment: A Scoping Review
Source: Nutrients. 2025 Oct 26;17(21):3365. doi: 10.3390/nu17213365 (PMC12610370; doi:10.3390/nu17213365)
Supplement: Supplementary file 1 [file nutrients-17-03365-s001.zip › nutrients-3874505-supplementary.pdf]

Supplementary file S1：Key Words Used in Searches

[MEDILINE(Pubmed)]

#1 ("Guibi-tang"[Title/Abstract] OR "Guibitang"[Title/Abstract]
OR "Kihi"[Title/Abstract] OR "Guipi"[Title/Abstract]
OR "Gui pi"[Title/Abstract] OR "帰脾"[Title/Abstract] OR "归脾"[Title/Abstract]) 73

#2 ("Cognitive"[Title/Abstract] OR "认知"[Title/Abstract] OR "認知"[Title/Abstract]) 541852

#3 ("Dementia"[MeSH Terms] OR "Dementia"[Title/Abstract]
OR "痴呆"[Title/Abstract] OR "失智"[Title/Abstract]) 286909

#4 ("Alzheimer Disease"[MeSH Terms] OR "Alzheimer"[Title/Abstract]
OR "アルツハイマー"[Title/Abstract] OR "阿尔茨海默"[Title/Abstract]) 227013

#5 #2 OR #3 OR #4 793957

#6 #1 AND #5 13

[Embase]

#1

귀비:ab,ti OR guibi:ab,ti OR kihi:ab,ti OR guipi:ab,ti OR "gui pi":ab,ti OR "帰脾 or 归脾":ab,ti

#2

인지 OR cognitive OR 认知 OR 認知 → 946,714

#3

치매 OR 'dementia'/exp OR dementia OR 痴呆 OR 失智 → 542,309

#4

알츠하이머 OR alzheimer OR アルツハイマー OR 阿尔茨海默 → 362,195

#5

#2 OR #3 OR #4 → 1,347,039

#6

#1 AND #5 → 14

[Cochrane Library]

#1 귀비 OR Guibi OR Kihi OR Guipi OR Gui-pi OR 帰脾 OR 归脾 → 44

#2 인지 OR Cognitive OR 认知 OR 認知 →110276

#3 치매 OR Dementia OR 癡呆 OR 失智 → 31940

#4 알츠하이머 OR Alzheimer OR アルツハイマー OR 阿尔茨海默 → 16640

#5 #2 OR #3 OR #4 → 127460
#6 #1 AND #5 → 13

[China National Knowledge Infrastructure (CNKI)]

(TKA=TKA="Guibi" OR TKA="kihi" OR TKA="Guipi" OR TKA="Gui-pi" OR TKA="帰脾" OR TKA="归脾") → 5533

(TKA="Cognitive" OR TKA="认知" OR TKA="認知") → 1101675

(TKA="Dementia" OR TKA="痴呆" OR TKA="失智") → 62477

(TKA="Alzheimer" OR TKA="アルツハイマー" OR TKA="阿尔茨海默") → 51707

(TKA=TKA="Guibi" OR TKA="kihi" OR TKA="Guipi" OR TKA="Gui-pi" OR TKA="帰脾" OR TKA="归脾") AND ((TKA="Cognitive" OR TKA="认知" OR TKA="認知") OR (TKA="치매" OR TKA="Dementia" OR TKA="痴呆" OR TKA="失智") OR (TKA="Alzheimer" OR TKA="アルツハイマー" OR TKA="阿尔茨海默")) → 100

[ScienceON]

#1 귀비 OR Guibi OR Kihi OR Guipi OR Gui-pi OR 帰脾 OR 归脾 → 661

#2 인지 OR Cognitive OR 认知 OR 認知 →1193226

#3 치매 OR Dementia OR 癡呆 OR 失智 → 273341

#4 알츠하이머 OR Alzheimer OR アルツハイマー OR 阿尔茨海默 → 304497

#5 #2 OR #3 OR #4 → 1517944
#6 #1 AND #5 → 47

[Scopus]

#1 귀비 OR Guibi OR kihi OR Guipi OR Gui-pi OR 帰脾 OR 归脾 → 165

#2 인지 OR Cognitive OR 认知 OR 認知 → 1106682

#3 치매 OR Dementia OR 痴呆 OR 失智 → 262185

#4 알츠하이머 OR Alzheimer OR アルツハイマー OR 阿尔茨海默 → 312330

#1 AND (#2 OR #3 OR #4) → 23

[Citation Information by National Institute of Informatics (CiNii)]

#1 귀비 OR Guibi OR kihi OR Guipi OR Gui-pi OR 帰脾 OR 归脾 → 248

#2 인지 OR Cognitive OR 认知 OR 認知 → 372569

#3 치매 OR Dementia OR 痴呆 OR 失智 → 76901

#4 알츠하이머 OR Alzheimer OR アルツハイマー OR 阿尔茨海默 → 82734

#5 #2 OR #3 OR #4 → 488005
#6 #1 AND # 5 → 16
